# Suppression of Proinflammatory Cytokines and Mediators in LPS-Induced RAW 264.7 Macrophages by Stem Extract of *Alternanthera sessilis* via the Inhibition of the NF-*κ*B Pathway

**DOI:** 10.1155/2018/3430684

**Published:** 2018-08-01

**Authors:** Katyakyini Muniandy, Sivapragasam Gothai, Khaleel M. H. Badran, S. Suresh Kumar, Norhaizan Mohd Esa, Palanisamy Arulselvan

**Affiliations:** ^1^Laboratory of Vaccines and Immunotherapeutics, Institute of Bioscience, Universiti Putra Malaysia, 43400 Serdang, Selangor, Malaysia; ^2^Department of Medical Microbiology and Parasitology, Faculty of Medicine and Health Sciences, Universiti Putra Malaysia, 43400 Serdang, Selangor, Malaysia; ^3^Department of Nutrition and Dietetics, Faculty of Medicine and Health Sciences, Universiti Putra Malaysia, 43400 Serdang, Selangor, Malaysia; ^4^Muthayammal Centre for Advanced Research, Muthayammal College of Arts and Science, Rasipuram, Namakkal, Tamil Nadu 637408, India; ^5^Scigen Research and Innovation Pvt. Ltd., Periyar Technology Business Incubator, Periyar Nagar, Thanjavur, Tamil Nadu 613403, India

## Abstract

*Alternanthera sessilis*, an edible succulent herb, has been widely used as herbal drug in many regions around the globe. Inflammation is a natural process of the innate immune system, accompanied with the increase in the level of proinflammatory mediators, for example, nitric oxide (NO) and prostaglandin (PGE_2_); cytokines such as interleukin 6 (IL-6), interleukin 1*β* (IL-1*β*), and tumor necrosis factor alpha (TNF*α*); and enzymes including inducible nitric oxide synthase (iNOS) and cyclooxygenase-2 (COX-2) via the activation and nuclear translocation of nuclear factor kappa-light-chain-enhancer of activated B cells (NF-*κ*B) subunit p65 due to the phosphorylation of inhibitory protein, I*κ*B*α*. Inflammation over a short period of time is essential for its therapeutic effect. However, prolonged inflammation can be detrimental as it is related to many chronic diseases such as delayed wound healing, cardiovascular disease, arthritis, and autoimmune disorders. Therefore, ways to curb chronic inflammation have been extensively investigated. In line with that, in this present study, we attempted to study the suppression activity of the proinflammatory cytokines and mediators as a characteristic of anti-inflammatory action, by using stem extract of *A. sessilis* in the lipopolysaccharide- (LPS-) stimulated RAW 264.7 macrophage cell line. The results showed that the extract has significantly inhibited the production of the proinflammatory mediators including NO and PGE_2_; cytokines comprising IL-6, IL-1*β*, and TNF*α*; and enzymes covering the iNOS and COX-2 by preventing the I*κ*B*α* from being degraded, to inhibit the nuclear translocation of NF-*κ*B subunit p65 in order to hinder the inflammatory pathway activation. These results indicated that the stem extract of *A. sessilis* could be an effective candidate for ameliorating inflammatory-associated complications.

## 1. Introduction

Health issues such as diabetic foot ulcer, pneumonia, Alzheimer, Parkinson's, and colitis are some of the complications that arise due to abnormalities of inflammatory responses taking place at a localized region or organ [1]. Besides, severe dysregulation of inflammation occurring systemically which is known as the sepsis is one of the prominent reason of mortality all over the world, and in Malaysia only, the Malaysian Registry of Intensive Care mentioned that it was the most common diagnosis leading to the admittance to the intensive care unit (ICU) in Health Ministry hospitals in 2011. However, the health problems occur only when the inflammation is prolonged and not regulated to its original state. Therefore, inflammation does not necessarily mean to cause destruction; in fact, inflammation is an essential natural response of our innate immune system upon any injury or exposure towards infectious agents in order to remove the harmful agents away [2].

Macrophage, the primary cell of the immune system, is an actively involved immune cell that acts by elongating its false feet to encourage phagocytosis of the agents to permit clearance to maintain an ideal environment for optimum body function [3]. With the major aim of inflammation is to get rid of the detrimental agents from our body, when a foreign material is present, it causes a specific binding protein on the body cell surface to fix and form a complex known as the pathogen-associated molecular patterns. The process starts with the detection of the binding complex by the Toll-like family receptors, specifically by TLR4 aided by the coreceptor, CD14 [4]. The binding action with TLR4 induces phosphorylation and eventually activates the nuclear factor kappa-light-chain-enhancer of the activated B cell (NF-*κ*B) inflammatory signaling pathway [5]. NF-*κ*B is a transcription factor that regulates the transcription of DNA to control the expression of protein-encoding genes for numerous biological processes. Due to the response with the existence of cellular stimuli, the inflammatory signaling is activated resulting in the transcribing proinflammatory genes thereby generating proinflammatory mediators and cytokines including nitric oxide (NO), cyclooxygenase-2 (COX-2), inducible nitric oxide synthase (iNOS), interleukin 6 (IL-6), interleukin 1*β* (IL-1*β*), tumor necrosis factor alpha (TNF*α*), and prostaglandin (PGE_2_) [6].

The upsurge of the proinflammatory products from the activated inflammatory pathway that are involved in the process of pathological pain is vital for a more profound immune response. Their accumulation itself may act in a positive feedback mechanism action that aggravates the release of more of the proinflammatory entities, as they can act as the inflammatory stimuli in activating a signaling pathway [7]. Over time, prolonged inflammation may cause damage to the tissues around the affected region. Habitually, the inflammatory-associated complicates arise when there is an impairment of the regulatory mechanism that consequences in the spontaneous development of chronic condition. Therefore, as the parameters of inflammation, the proinflammatory mediators and cytokines should be downregulated by targeting the transcription factor NF-*κ*B pathway to reduce the likeliness of pathological damage and pain [8].

In order to overcome the problem, there are countless range of effective anti-inflammatory treatments available, such as the NSAID and the statins. However, Singh et al. reported that NSAID may increase the risk of developing gastrointestinal tract complications that may lead to stomach and intestinal bleeding [9]. In line with that, the pharmaceutical industry still facing challenges in developing a more effective and lesser toxic drug that could be able to cater diverse groups of patients as the presently accessible drugs may be unsuitable to all patients according to their age and health background. Based on that, the necessity for the exploration for a better anti-inflammatory therapeutic agent is always in need.

In this regard, an edible plant, *Alternanthera sessilis*, was chosen as a possible candidate to test for its anti-inflammatory action. *A. sessilis* also known as kermak putih, bunga-bunga, or daun tolod in Malaysia is a succulent herb with the height of about 0.4 to 1.4 meters high [10]. The plant was an extremely valued vegetable and was reported that it has been used as herbal drugs in many regions around the globe with the reference of their past folklore practices. Complications such as diarrhoea, dysentery, wounds, fever, vomiting blood, headache, vertigo, neuralgia, hernia, hepatitis, tight chest, bronchitis, and asthma have been treated using this herb. It was also used as hair tonic, cholagogue, abortifacient, and galactagogue [11]. An earlier work that involved chromatography analysis has reported that the herb consists of anti-inflammatory-associated compounds such as catechin, rutin, ellagic acid, quercetin, vanillic acid, gallic acid, epicatechin, p-coumaric, ferulic acid, chlorogenic acid, ethyl gallate, 4-hydroxybenzoic, apigenin, and daidzein [12]. Besides, the leaves of *A. sessilis* were reported to suppress inflammation in the carrageenan-induced rat paw edema animal, however leaving a gap of research in the action mechanism of inflammatory parameters by targeting a specific signalling pathway [13]. Furthermore, not only the leaves of *A. sessilis* but the stem part was also proven to contain bioactive compounds through chromatography analysis and demonstrated to induce wound healing in skin cell lines [14]. Therefore, in this present study, we attempted to analyse the ability of the uncommon part of this plant, which is the stem, in the reduction of proinflammatory cytokines and mediators by targeting the NF-*κ*B inflammatory pathway on the RAW 264.7 macrophage cell line.

## 2. Materials and Methods

### 2.1. Cell Maintenance and Culture

RAW 264.7 murine macrophage cell line, purchased from American Type Culture Collection (ATCC, USA), was cultured in Dulbecco's modified minimal essential medium (DMEM) supplemented with 10% (*v*/*v*) of heat-inactivated fetal bovine serum and 1% antibiotics (100 U/mL penicillin and 100 *μ*g/mL streptomycin) and incubated at 37°C in a humidified incubator with 5% CO_2_. Cell that reached 80% confluency was subcultured and/or used for further experiments. The process of cell detachment involves trypsinization using TrypLE Express Enzyme (Gibco).

### 2.2. Preparation of Stem Extract of *A. sessilis*

The perennial herb was acquired from a market at Serdang, Selangor, Malaysia, and authenticated by Dr. Shamsul Khamis from the Biodiversity Unit, IBS, UPM (Malaysia), with the voucher specimen number of (SK 2938/15). The stem part of the vegetable was detached, washed, air-dried thoroughly, and grinded finely into a powder form. The finely grounded powder was macerated into 90% ethanol with the ratio of 9 : 1 (absolute ethanol : water) for 3 days at room temperature. The mixture was filtered using the filter paper, and the filtrate was furthered for evaporation to remove the solvent through a rotary evaporator. The thicken product was freeze dried and stored at −20°C until further usage for experiment.

### 2.3. Cell Viability Assay

RAW 264.7 murine macrophage cell line was plated into a 96-well plate at a density of 1 × 10^4^ in 100 *μ*L of DMEM medium per well and incubated overnight until it reaches confluence on the following day for treatment. In the course of treatment, the medium was replaced with various concentrations of stem extract including 25, 50, 100, 200, 300, 400, and 500 *μ*g/mL, and it was incubated for another 24 h. Following the 24 h of incubation with the treatments, 10 *μ*L of 5 mg/mL MTT solution was added into each well and incubated for another 4 h at 37°C. At that time, the plate was centrifuged and the solution was discarded leaving the crystal at the bottom of the plate. Then, 100 *μ*L dimethyl sulfoxide was added to dissolve the insoluble formazan salt. The plate was swirled gently and kept in a dark place for about 30 min. A microplate reader at the absorbance value of 570 nm was used to quantify the formazan formed. Finally, the percentage of cell viability (CV) was calculated by substituting the absorbance reading using the formula below:

(1) $$\begin{array}{c} CV\;\left(\%\right)=\frac{Absorbance\text{ of the }test\;sample}{Absorbance\text{ of }control}\times 100 \end{array}$$

Accordingly, the graph of percentage of cell viability of RAW 264.7 cells against concentrations of stem extract of *A. sessilis* was plotted. Experiments were performed in triplicate, and the data were presented as mean ± SD (*n* = 3).

### 2.4. Quantifying the Nitric Oxide Production

The generation of nitric oxide was determined by quantifying the production of the nitrite level by the Griess reaction. Firstly, the procedure involved the seeding of RAW264.7 cells into 6-well plates with the density of 1 × 10^6^ cells/well. On the next day, when the cells reach confluence, the cells were treated with different concentrations of stem extract of *A. sessilis* (50, 100, and 200 *μ*g/mL) and dexamethasone (0.5 *μ*g/mL) with another two more wells left untreated. After 2 h, all treated wells and an untreated well were supplemented with LPS (1 *μ*g/mL) to encourage inflammation. An untreated well without LPS remained as the control group. Following incubation for 24 h, the culture medium was collected and assayed for the nitrite level using a Griess reagent, which involved the solution mixture of (1% sulfanilic acid, 0.1% N-1-naphthyl-ethylenediamine dihydrochloride, and 5% phosphoric acid). Upon addition, the mixture was stored for 10 min in the dark condition and the absorbance was measured at 540 nm. The nitrite concentration of the sample was determined by comparing its absorbance with the sodium nitrite's which is the standard for NO_2_.

### 2.5. Measurement of Proinflammatory Cytokine Production

The analysis of proinflammatory cytokines was quantified using the supernatant collected from the treated cells, just like for the nitric oxide analysis. However, the analyses were conducted using ELISA (R&D Systems), and the protocol was based on the manual provided in the kit purchased. The measurement of the level of cytokines involved the transferring of the collected cell culture supernatant into four separate 96-well plates coated with the capture antibody against the respective cytokine provided in the kit. The plates were read by using a microplate spectrophotometer at the absorbance of 450 nm. Accordingly, the graph of the level of cytokines against concentrations of treatment was plotted.

### 2.6. Nuclear Translocation of NF-*κ*B p65 Measurement of Proinflammatory Cytokine Production

Investigation of protein location in a cell was performed using immunocytochemistry technique whereby it involved the fluorescent detection in which a fluorophore conjugated to the antibody that can be visualized using a fluorescence or confocal microscope. The cells were plated on cover slip in a 6-well plate. After 24 h of incubation with treatment, the cell culture medium was removed and the cells were washed gently with PBS and fixed with chilled paraformaldehyde. Then, the cells were permeabilized with Triton X-100. After permeabilization, the adhered cells were washed and blocked before being incubated with primary antibody of anti NF-*κ*B p-65 with the dilution for overnight at 4°C. On the following day, the wells were washed before the incubation of fluorophore-conjugated secondary antibody for 1 h at room temperature. Then, the washing step was repeated, and the cells were incubated with Hoechst dye for 10 min. Subsequently, the cells were washed again, and the cover slips were removed and mounted on a glass slide with a gold antifade reagent sealed with crystal clear nail polish. The slides were viewed under a confocal microscope with the magnification of ×600, and images were captured.

### 2.7. Protein Expression Analysis

After 24 h of treatment and incubation, the cells were collected by scraping the cells using a scraper. The cells we centrifuged into pellet and ice cold RIPA mammalian protein extraction lysis buffer with protease, and phosphatase inhibitor cocktails (Roche, Basel, Switzerland) were added and centrifuged to extract the protein from the supernatant of the cell. The total concentration of protein was determined using a bicinchoninic acid (BCA) protein assay kit with bovine serum albumin (BSA) as the standard. The analysis of protein expression was identified by conducting Western blot. Primarily, equal concentration of the protein sample was loaded and separated by sodium dodecyl sulfate polyacrylamide gel electrophoresis (SDS-PAGE). A protein marker was also loaded to indicate molecular weight. The gel was blotted onto the polyvinylidene fluoride (PVDF) membrane. The membrane was then blocked with 5% BSA before being with specific antibodies and incubated overnight at 4°C. On the following day, the membrane was washed and incubated with suitable HRP-conjugated secondary antibodies for 1 h at room temperature. In the following incubation, the membrane was washed and chemiluminescence substrate (Thermo Scientific, Rockford, IL, USA) was added just before viewing the blots. They were viewed using a ChemiDoc™ XRS (Bio-Rad).

### 2.8. Liquid Chromatography-Mass Spectrometry (LC-MS)

The nonvolatile compounds in the ethanolic extract of *A. sessilis*'s stem were identified using the AB Sciex 5500 QTrap LC-MS/MS with Agilent Technologies 1290 series UHPLC. The extract was diluted in equal volume of methanol and water. The separation was performed using the column Phenomenex Synergi Fusion 100 mm × 2.1 mm × 3 *μ*m held at 25°C with the enhanced MS (EMS) parameters as follows: nebulizer gas (N2) temperature 500°C, flow rate 10 L/min, pressure 40 psi, and capillary voltage 4500 V, with the scanning range of 100–1000 *m*/*z* for full scan and 50–1000 *m*/*z* for MS/MS scan. The fragmentor voltage was 135 V, and injection volume for all samples was 10 *μ*L; they were 100–1000 *m*/*z* for full scan and 50–1000 *m*/*z* for MS/MS scan. Solvent A was water with 0.1% formic acid and 5 mM ammonium formate, and solvent B was acetonitrile with 0.1% formic acid and 5 mM ammonium formate. The gradient elution was performed as follows: 5% B to 95% B from 0.01 min to 10.0 min, hold for 2 min and back to 10% B in 0.1 min, and reequilibrated for 3 min.

### 2.9. Statistical Analysis

Results obtained were analyzed using SPSS software one-way analysis of variance (ANOVA) followed by a post hoc Tukey's test. The experiments were performed in triplicate, and the data were reported as the mean ± standard deviation (SD); these values were obtained from triplicate experiments. ^###^*p* < 0.001, the LPS-treated group versus the control group; ^∗∗∗^*p* < 0.001, ^∗∗^*p* < 0.01, and ^∗^*p* < 0.05, the treated group significantly different from the LPS-treated group. Besides, the differences among the groups were considered statistically significant when the *p* values were 0.05 or less.

## 3. Results

### 3.1. Cytotoxic Effect of Stem Extract of *A. sessilis* on the Viability of RAW 264.7 Cells

**Figure 1:**
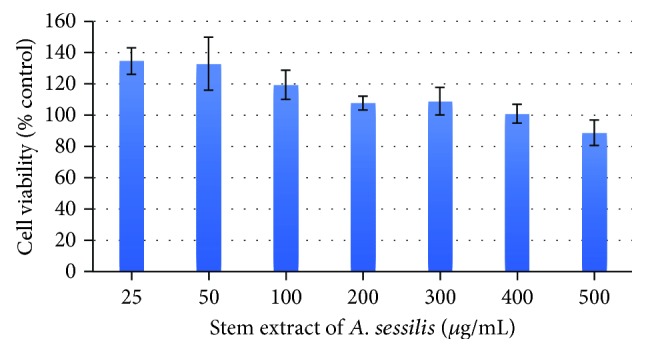
Effect of stem extract of *A. sessilis* on the cell viability on the RAW macrophage cell line. The cell viability was expressed as the percentage compared with the untreated (control) cell group. The error bars were added into the plot to signify the standard deviation between triplicates.

Selection of suitable nontoxic range of extract concentrations is important for further anti-inflammatory analysis. Therefore, the cytotoxicity of stem extract of A. sessilis was evaluated by measuring the percentage of cell viability on RAW 264.7 macrophages using MTT reduction assay for 24 h. As shown in Figure 1 below, a slight reduction in cell viability was noticeable as the concentration increases, yet it was clearly portrayed that even at the highest concentration (500 *μ*g/mL), the percentage of cell viability remained above 80% so it is a noncytotoxic dosage. The concentrations of 200 to 400 *μ*g/mL showed a very minor changes in cell viability; meanwhile, the concentration 500 *μ*g/mL showed relatively large standard deviation thereby it is high risk to choose this concentration as it may fall into the toxic dosage grade. Therefore, safer concentrations which were 50, 100, and 200 *μ*g/mL were chosen as the nontoxic range of treatment for RAW 264.7 macrophages for further anti-inflammatory analysis.

### 3.2. The Antinitric Oxide Production Effect of Stem Extract of *A. sessilis* in LPS-Stimulated RAW 264.7 Cells

**Figure 2:**
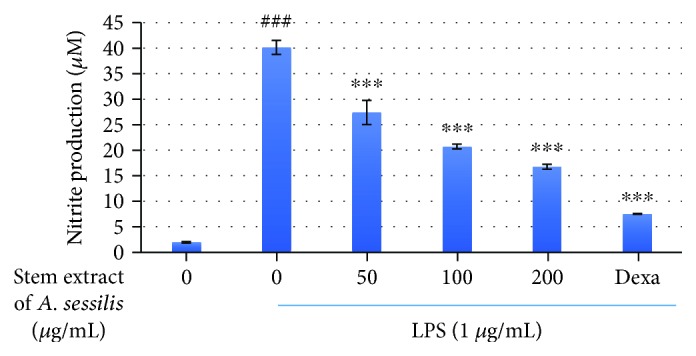
The effect of stem extract of A. sessilis on NO production in LPS-stimulated RAW 264.7 macrophage cells. The nitric oxide level was studied by observing the nitrite level production by Griess reagents. The values of nitrite production were expressed in *μ*M, and the data presented were the mean values of three experiments ± S.D. ^###^*p* < 0.001, the LPS-treated group versus the control group; ^∗∗∗^*p* < 0.001, the treated group significantly different from the LPS-treated group.

Nitric oxide is a major mediator in the persistence of inflammation that contributes to its pathogenicity. Hence, to control its production is a principal role in an anti-inflammatory investigation. The level of nitric oxide is measured by using a Griess reaction test whereby the nitrite ion contained in the sample was quantified by comparing its absorbance with the standard prepared.  Figure 2 showed that the normal macrophage produces a minute amount of nitric oxide. Stimulation with LPS induced inflammation and have prompted the NO production with the nitrite level peaking to 40 *μ*M. Upon treatment with the stem extract of *A. sessilis*, the NO level was reduced in a dose-dependent manner. The highest concentration of extract has suppressed the production of nitric oxide of about 2 folds, when compared to the LPS-stimulated group.

### 3.3. Inhibitory Effect of Stem Extract of *A. sessilis* on the Expression of Proinflammatory Cytokines in LPS-Stimulated RAW 264.7 Cells

**Figure 3:**
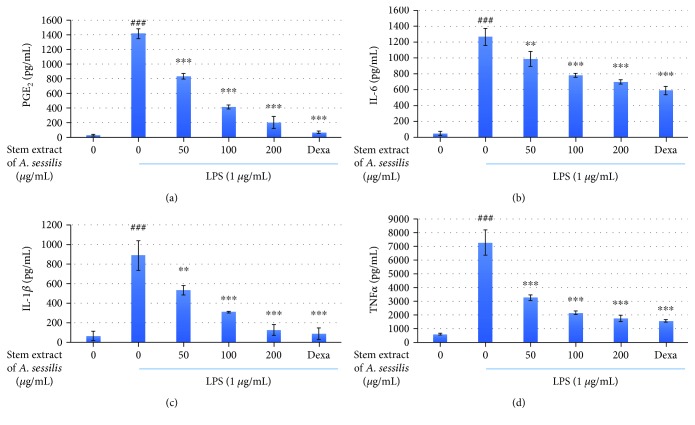
The effect of stem extract of A. sessilis on prostaglandin E2 (PGE2) (a), interleukin IL-6 (b), IL-1*β* (c), and tumor necrosis factor-*α* (TNF*α*) (d) in LPS-stimulated RAW 264.7 macrophage cells. The values of cytokine production were expressed in pg/mL, and the data presented were the mean values of three experiments ± S.D. ^###^*p* < 0.001, the LPS-treated group versus the control group; ^∗∗∗^*p* < 0.001 and ^∗∗^*p* < 0.01, the treated group significantly different from the LPS-treated group.

PGE_2_, IL-6, IL-1*β*, and TNF*α* are some of the proinflammatory cytokines that participated in the prolonging of chronic inflammation. These cellular messengers should be mitigated as a measure to avoid further inflammatory process. The level of the cytokine production in LPS-induced macrophages was evaluated using ELISA technique. Exposure of the cells with LPS had significantly increased the production of PGE_2_ (a), IL-6 (b) IL-1*β* (c), TNF*α* (d) in Figure 3 up to 48, 25, 13, and 11 folds, respectively, as compared to their respective control groups. However, the treatment with stem extract of *A. sessilis* had suppressed the production of the cytokines significantly in a dose-dependent manner. The inhibition of the proinflammatory cytokine activity of the highest concentration of extract (200 *μ*g/mL) was noted to be with the fold change of 6, 1, 6, and 3 in the order, in comparison with dexamethasone groups (positive control-treated group) which were 20, 1, 9, and 5 folds, respectively, when they were compared one-to-one to its respective LPS-treated group. Among the four types of proinflammatory cytokines, the reduction of IL-6 by stem extract of *A. sessilis* was noted to exhibit the closest inhibitory activity with the dexamethasone group.

### 3.4. Suppression Effect of Stem Extract of *A. sessilis* on Nuclear Translocation of the Unit of NF-*κ*B Subunit p65 in LPS-Stimulated RAW 264.7 Cells

**Figure 4:**
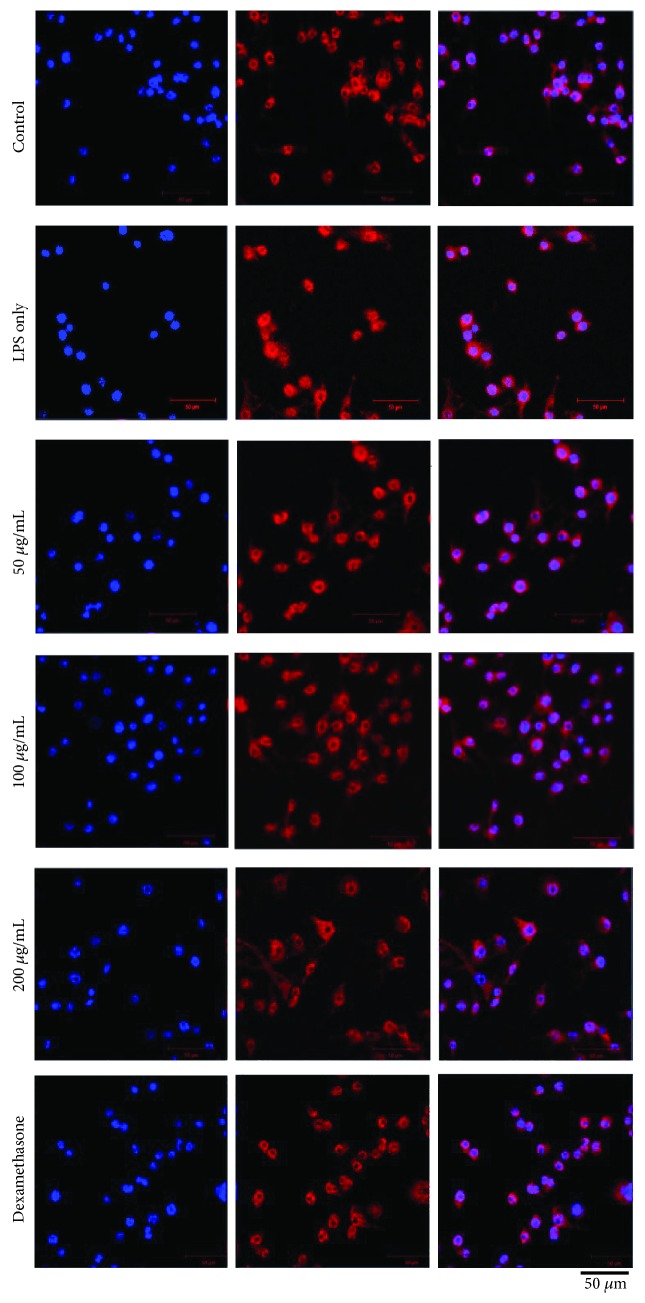
Effect stem extract of *A. sessilis* on the nuclear translocation of the unit of NF-*κ*B in LPS-stimulated RAW264.7 macrophages. The treated cells were fixed and processed for immunostaining with specific antibodies. The nucleus was stained with Hoechst dye (blue color), and NF-*κ*B subunit p65 (red color) was observed with a fluorescent microscope with the magnification of images, ×600.

**Figure 5:**
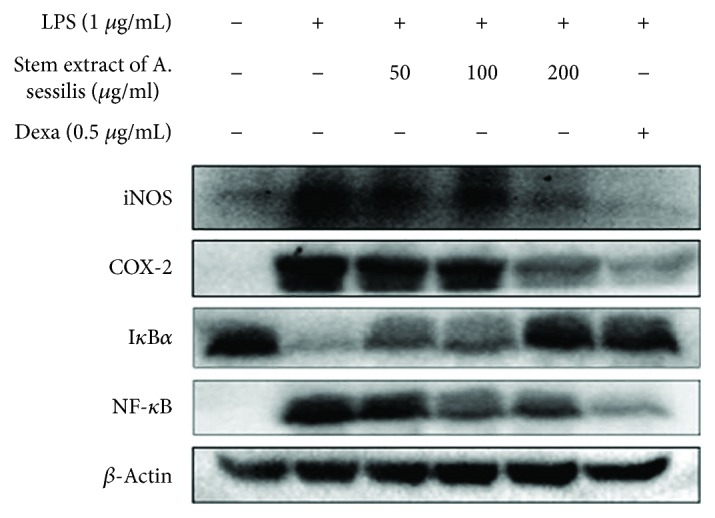
Effect of stem extract of *A. sessilis* on the protein expression of inflammatory enzymes, cyclooxygenase- (COX-) 2, inducible nitric oxide synthase (iNOS), inflammatory regulator, nuclear factor NF-*κ*B p65, and inhibitor of *κ*B (I*κ*B*α*) on the macrophage induced with LPS and treated with various concentrations of *A. sessilis* stem extract (50, 100, and 200 *μ*g/mL) for 24 h. Protein was extracted from the cells and furthered to electrophoresis, and protein expression was detected by Western blots according to its appropriate antibodies. The experiments were carried out in triplicate, and images shown are representatives of triplicates.

Activation of the inflammatory signaling pathway takes place when the NF-*κ*B subunit p65 translocates from cytoplasm to the nucleus. Therefore, analysis to identify the location of the subunit p65 is essential to verify the effect of treatment towards the action mechanism. The degradation of I*κ*B*α* releases and activates the NF-*κ*B p65 to be translocated from cytoplasm into the nucleus. This forms an activated form of the transcription factor that begins to transcribe in the inflammatory mediators. However, the stem extract of *A. sessilis* was demonstrated to inhibit the translocation of the NF-*κ*B subunit p65 to the nucleus as noted that the intensity of NF-*κ*B p65 (red stain) reduces in a dose-dependent manner (Figure 4). Besides, parallel experiments such as inflammatory cytokines (Figure 3) and inflammatory markers (Figure 5) noted to demonstrate a similar pattern of reduction.

### 3.5. The Effect of Stem Extract of *A. sessilis* on the Protein Expression in LPS-Stimulated RAW 264.7 Cells

The NF-*κ*B signaling pathway is an important regulator in the manufacture of inflammatory cytokines and markers. iNOS and COX-2 are the proinflammatory enzymes responsible in the production of NO and PGE_2_, respectively. Inflammation induced the expression of both enzymes by the activation of NF-*κ*B as shown in Figure 5, which was upregulated after inflammation. The expression pattern of iNOS and COX-2 was similar with the production of NO (Figure 2) and PGE_2_ (Figure 3(a)). Treating the cells with stem extract of *A. sessilis* noted to reduce the expression of iNOS and COX-2, and it occurred to be parallel with the reduction effect for the production of NO and PGE_2_. This was due to the downregulation of NF-*κ*B. However, the expression of I*κ*B*α* was antagonistic with the other protein in the study. This is due to its role as an inhibitor for the activation of the signaling pathway. In a normal/resting cell, the stable I*κ*B*α* inhibits cytoplasm-residing NF-*κ*B p65 in an inactive form. LPS which stimulates cascade effects phosphorylated I*κ*B*α* thereby it loses its structure and degrades, which in turn releases NF-*κ*B p65 free to be translocated into the nucleus to initiate transcription of the proinflammatory genes. Nevertheless, the pretreatment of stem extract of *A. sessilis* reversed the effect by preventing the phosphorylation of I*κ*B*α* and at the same time preventing the activation NF-*κ*B p65 in a dose-dependent manner.

### 3.6. Compound Identification in the Stem Extract of *A. sessilis* by Liquid Chromatography-Mass Spectroscopy (LC-MS)

**Figure 6:**
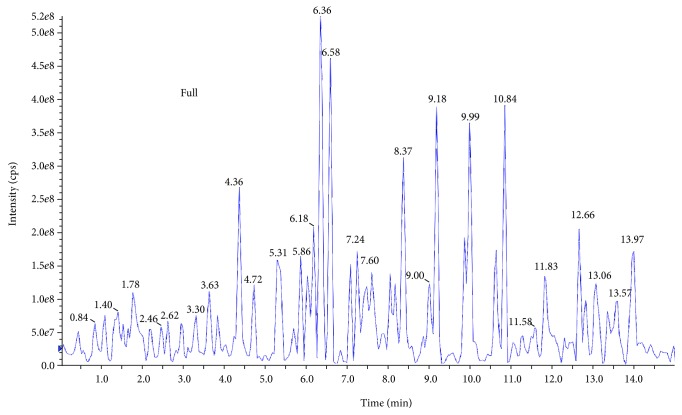
Liquid chromatogram for stem extract of *A. sessilis.*

The results attained from LC-MS analysis of the stem extract of *A. sessilis* showed the existence of 27 noticeable peaks in the chromatogram (Figure 6). Analysis of the mass spectra was carried with the databases such as the MassBank and KNApSAcK Core DB. However, only 9 spectra were known for its possible compound. According to its previous biological study, 7 of the identified compounds have demonstrated anti-inflammatory activity. Besides, antioxidant and antimicrobial properties were also reported in the identified compounds.

## 4. Discussion

The results of the present investigation indicated that the ethanolic extract of stem of *A. sessilis* exhibited anti-inflammatory properties by quashing the production of proinflammatory cytokines and mediators in LPS-stimulated macrophage; therefore, the hypothesis of the study was accepted. Inflammation is the generic manner response by the innate immune system towards the presence of foreign agents such as bacteria. Commercially available LPS that is used to stimulate inflammation is an endotoxin isolated from the gram-negative bacteria [4]. It is used to mimic the bacterial invasion as in the real scenario of bacterial infection; the immune system lyses the bacteria and releases the lipid portion of the LPS into the body system that initiates the inflammatory process. Often, in proof-of-concept analysis of any biological activity in an extract or a compound, the pretreatment method is encouraged before LPS is added as it demonstrates a clearer result. This is mainly due to the sticky nature of the LPS to retain its structure that may interfere with the sample being tested; therefore, they were added separately [34].

According to the correspondence by the innate immune cells, LPS activates the signaling pathway such as NF-*κ*B via the stimulation of Toll-like receptor 4 (TLR4) which consequently releases proinflammatory cytokines including IL-1B, TNF*α*, and IL-6 and mediators such as NO and PGE_2_ [1] which marks the occurrence of inflammation. The cytokines as well as the mediators play a vital role in inflammation by its chemotactic and vasoactivator properties by increasing the permeability of the blood vessel for cells and fluid and recruiting more inflammatory cells to the affected region, respectively. Its primary goal is to provide localization at the infected area and to remove the detrimental agent and the impaired tissue to facilitate a healing process. Cytokines and mediators are extremely important for the maintenance of optimum inner environment of our system, from injurious agents. However, they should be regulated back into its original level as their elevated amount for a prolonged duration of time may develop into chronic inflammation that is detrimental. This is due to the phenomena whereby the cytokines and mediators themselves may act as a stimulus in activating the inflammatory pathway.

The LPS-stimulated macrophage induces a large assembly of nitric oxide, which is synthesized from L-arginine by the enzymatic action of NO synthase (NOS) specifically inducible NOS (iNOS) which is expressed excessively during an inflammation. iNOS is the key enzyme is the production of NO. Furthermore, the LPS-induced macrophage also increases the production of PGE_2_ which is associated with the occurrence of pathological pain during inflammation. The generation of prostaglandin-endoperoxide synthase 2 (PGE_2_) is responsible of the bioactivity of cyclooxygenase-2 (COX-2); therefore, its upregulation during inflammation results in the increased production of PGE_2_ [35]. Pretreatment of stem extract of *A. sessilis* suppressed the expression of iNOS and COX-2 which causes the decrease in the production of NO and PGE_2_, respectively. The reduction of NO is also associated with the reduction of oxidative stress by the antioxidative system, regulated by the antioxidative compounds present in the place such as apigenin-6,8-di-C-*β*-D-glucopyranoside isomer, kaempferol monosulfate, and p-hydroxybenzoic acid which were previously reported for its antioxidant activity [36]. PGE_2_ is a key mediator for the sensitization of pathological pain by intensifying the receptiveness of peripheral nociceptors in irritated tissues. At the same time, the 2^″^-O-rhamnosylvitexin, apigenin-6,8-di-C-*β*-D-glucopyranoside isomer, kaempferol monosulfate, and p-hydroxybenzoic acid identified in the extract as shown in Table 1 were also found to possess natural antinociceptive activity therefore, aid in the blocking of the detection of pain stimuli by sensory neurons.

Additionally, cytokines which are the small sized proteins that function as the messenger between cells also play an important role during inflammation. Intereukin-6, interleukin 1*β*, and tumor necrosis factor-*α* are some of the cytokines that are elevated in LPS-induced macrophage to combat the infection. The pleotropic nature of the protein explains that the accumulation of a high level of proinflammatory cytokines amplifies inflammation due to the complexity of the interdependency of one on the other as the production of proinflammatory cytokines that work in a cascade mechanism by downstream effects of earlier released cytokines [37]. For examples, IL-1 stimulates TNF and IL-6 [38, 39]; meanwhile, the PGE_2_ promotes the release of IL-6 [40]. Therefore, suppressing the production of a cytokine may influence the reduction of another dependent cytokine. Accordingly, in this analysis, the stem extract was able to quash the level of IL-6, IL-1*β*, and TNF*α* in a dose-dependent manner.

The investigation conducted showed that the stem extract of *A. sessilis* was capable of suppressing the proinflammatory molecules thereby providing us a concrete justification that this extract has a potential value to be carried forward for a more advanced anti-inflammatory studies. However, the suppression activity is incomplete without its inhibitory mechanism study. In line with that, the NF-*κ*B transcription family that regulates the initiation of transcription of proinflammatory genes was included in the experiment. It is a key modulator of inflammation that operates by the nuclear translocation of cytoplasmic complexes. In a normal/resting state without the presence of any threat, the NF-*κ*B subunit p65 is located in the cytoplasm in an inactive form allied with an inhibitory protein, I*κ*B*α*. On the other hand, when threat such as LPS is detected, a cascade reaction takes place that results in the phosphorylation and degradation of I*κ*B. The complex degradation consequence is the release of the cytoplasmic NF-*κ*B-I*κ*B*α* complex to be translocated into the nucleus and binds to the enhancer elements of target genes thereby encouraging the transcription of the target proinflammatory genes.

The protein expression study in Figure 5 clearly showed that NF-*κ*B increased to its highest intensity after stimulation with LPS and reduced in a dose-dependent manner after treatment with the stem extract of *A. sessilis*. The inhibition of the NF-*κ*B protein expression showed a similar pattern of reduction for the proinflammatory enzymes, COX-2 and iNOS; the mediators, PGE_2_; and cytokines, IL-1*β*, TNF*α*, and IL6. Conversely, antagonistic effect was observed in the expression I*κ*B*α* whereby it was at its highest intensity in control when it was intact, but LPS stimulation causes structural degradation; therefore, it was at its lightest intensity in the LPS-induced group of treatment. However, upon treatment with our extract, it marks the increase in the band intensity as the dose of extract increases which concludes that the stem extract of *A. sessilis* conserves the structural stability of the inhibitory protein, I*κ*B*α*, with the main focus to keep the NF-*κ*B signaling pathway dormant.

**Figure 7:**
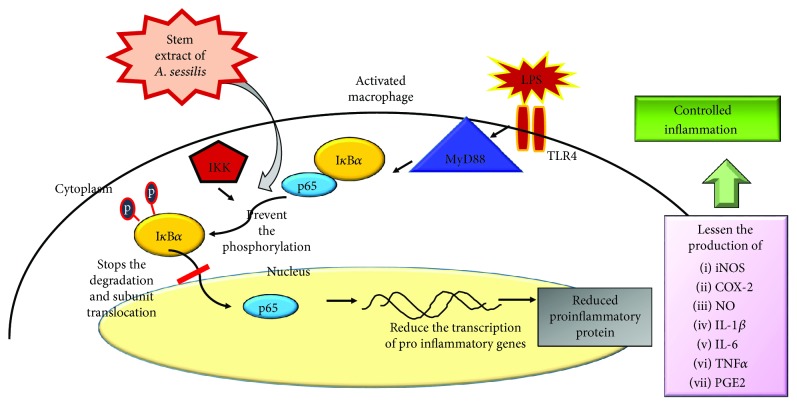
Image above shows the NF-*κ*B inflammatory signaling pathway. The stem extract of *A. sessilis* has inhibited the phosphorylation, ubiquitination, and degradation of the inhibitory protein, I*κ*B*α* complex. Hence, the NF-*κ*B subunit p65 stayed intact with the inhibitory protein, stopping the cytoplasm-residing NF-*κ*B subunit p65 from being translocated to the nucleus to activate the proinflammatory gene expression.

Moreover, the location of the NF-*κ*B subunit p65 was identified by immunocytochemistry analysis (Figure 4) with the aid of blue nuclear stain and NF-*κ*B p65 antibody conjugated with a fluorescent probe which is red in color. The blue and red stain signifies the translocation of NF-*κ*B p65 into the nucleus. Accordingly, migration of NF-*κ*B p65 was demonstrated in the LPS-stimulated group; meanwhile; in the treatment groups, our extract reduces the degree of migration that results in the activation of the NF-*κ*B signaling pathway. Hence, the result validated that the stem extract of *A. sessilis* has repressed the generation of proinflammatory cytokines and mediators by hindering the degradation of I*κ*B*α* to inhibit the initiation of the NF-*κ*B pathway as shown in Figure 7.

## 5. Conclusions

In summary, the stem extract of *A. sessilis* has effectively and efficiently reduced the level of proinflammatory cytokines and mediators in LPS-stimulated RAW 264.7 macrophages by inactivating their corresponding genes at the transcriptional level and by preventing the activation of the NF-*κ*B pathway that was achieved by obstructing the phosphorylation of I*κ*B*α*. Therefore, based on the finding, stem extract of *A. sessilis* may thus be considered as an impending anti-inflammatory therapeutic agent against inflammatory-associated problems. Even so, the *in vitro* analysis is insufficient to be directly extrapolated its biological activity; therefore, animal studies should be included to study its bioactivity, molecular mechanism(s), and drug distribution in an inflammation-induced animal model.

## Figures and Tables

**Table 1:** Total compounds identified in the crude extract of the stem part of *A. sessilis* using LC-MS analysis in a negative mode with its molecular mass, fragmentations, and reported biological activities.

Compounds	MW	Reported biological activities	Citations
2^″^-O-rhamnosylvitexin	577.04	Antihyperalgesic	[15–18]
		Anti-inflammatory	
		Antinociceptive	
		Prothrombolytic	
		Antidiabetic	

Apigenin-6,8-di-C-*β*-D-glucopyranoside isomer	594.05	Antioxidant	[19–22]
		Anti-inflammatory	
		Antinociceptive	
		Anticancer Antitumor	
		Hepatoprotective	
		Antidiabetes	
		Antiviral	
		Antibacterial Antifungal	
		Diabetic wound healing	

Kaempferol monosulfate	364.98	Antioxidant	[19, 20, 23, 24]
		Anti-inflammatory	
		Antinociceptive	
		Wound healing	
		Antibacterial	

p-Hydroxybenzoic acid	137.02	Antioxidant	[25–28]
		Wound healing	
		Analgesic	
		Anti-inflammatory	
		Neuroprotective	
		Antibacterial	
		Antifungal	
		Demelanizing agent	

p-Hydroxycinnamoyl moiety	164.08	Antibacterial	[28]
		Antifungal	
		Demelanizing agent	

Protocatechuic acid	153.02	Antioxidant	[19, 27]
		Anti-inflammatory	
		Neuroprotective	

Gibberellin	330.89	Antiulcer	[29, 30]
		Wound healing	
		Anti-inflammatory	

Daidzein	253.16	Anti-inflammatory	[31, 32]
		Antilipogenesis	

Benzophenone-4	307.09	Endocrine-disrupting activity	[33]

MW: molecular weight.

## Data Availability

The data used to support the findings of this study are available from the corresponding author upon request.
